# Kolmogorov Basic Graphs and Their Application in Network Complexity Analysis

**DOI:** 10.3390/e23121604

**Published:** 2021-11-29

**Authors:** Amirmohammad Farzaneh, Justin P. Coon, Mihai-Alin Badiu

**Affiliations:** Department of Engineering Science, University of Oxford, Oxford OX1 4BH, UK; amirmohammad.farzaneh@eng.ox.ac.uk (A.F.); mihai.badiu@eng.ox.ac.uk (M.-A.B.)

**Keywords:** graphs, Kolmogorov complexity, graph complexity, networks

## Abstract

Throughout the years, measuring the complexity of networks and graphs has been of great interest to scientists. The Kolmogorov complexity is known as one of the most important tools to measure the complexity of an object. We formalized a method to calculate an upper bound for the Kolmogorov complexity of graphs and networks. Firstly, the most simple graphs possible, those with O(1) Kolmogorov complexity, were identified. These graphs were then used to develop a method to estimate the complexity of a given graph. The proposed method utilizes the simple structures within a graph to capture its non-randomness. This method is able to capture features that make a network closer to the more non-random end of the spectrum. The resulting algorithm takes a graph as an input and outputs an upper bound to its Kolmogorov complexity. This could be applicable in, for example evaluating the performances of graph compression methods.

## 1. Introduction

Kolmogorov complexity (K-complexity) [1], of a string of characters, is the length of the shortest possible program that, when fed into a universal Turing machine, outputs that string. This concept, since it was introduced by Kolmogorov, has been used as a measure of complexity and randomness [2]. The Kolmogorov complexity of a string is incomputable [3], but upper bounds to it can be found [4]. Having a more accurate estimate of the upper bound of the Kolmogorov complexity is of great importance, as it could help in areas, such as data compression and data mining [5,6].

Graphs are considered some of the most important data structures in mathematics and physics. They are used to simulate networks and they have major applications in various areas, such as communication systems [7], social sciences [8], earth sciences [9], and biology [10]. However, analyzing graphs has always been a challenge, due to the complex nature of these data structures. To date, there have been numerous attempts to capture and measure the complexity of graphs [11]. These attempts have used different methods to provide a complexity measure for networks. [11] divides these approaches into two categories: deterministic and probabilistic. K-complexity and Shannon’s entropy [12] are the dominant approaches in the deterministic and probabilistic domains, respectively. At first sight, entropy may seem to be a better approach for calculating the complexity of a random variable, especially as a metric for graph compressibility. Notice that entropy is defined for an ensemble, and it needs a probability distribution defined on the random variable at hand. However, when studying graphs, we are usually faced with a single one rather than an ensemble, and the probability distribution is barely ever known. Additionally, [13] mentions that K-complexity is a more robust and reliable measure of network complexity rather than entropy, and [14] discusses the fact that entropy might be a deceiving measure for the complexity of graphs. Consequently, we use Kolmogorov’s metric for measuring the complexity of graphs, and will present a more accurate image of the K-complexity of individual graphs.

A simple graph on *n* vertices can be fully represented using a sequence of n2 bits, which can be seen as a reshaping of the graph’s adjacency matrix. As a result, n2 is often seen as the upper bound for the K-complexity of all graphs with *n* nodes [11]. Additionally, for some graphs, other representation methods, such as adjacency lists, might result in codes shorter than n2 bits, which will result in a lower upper bound for the K-complexity of these graphs. Ref. [15] obtains a canonical code for the structure of a graph by reordering the rows and columns of its adjacency matrix, as an estimate of its K-complexity. From a more general viewpoint, the code word length of any lossless compression method for graphs can be considered as an upper bound to the K-complexity of graphs [16]. There exist numerous compression and summarization algorithms that focus on graphs ([17,18]), but these algorithms are not directly tested for calculating the complexity of graphs. Other methods have a more general approach and utilize the notion of algorithmic probability to feed random programs into a Turing machine and count the number of times that the machine produces the desired output [13,16]. However, we believe that a formalized method for estimating the K-complexity of graphs based on their structural properties is needed. Our proposed algorithm takes on a generative approach, similar to the concept and calculus introduced in [19,20], but with a focus on simple graphs.

In this paper, a novel approach to the K-complexity of graphs is presented. We search for the least complex graphs possible on *n* nodes in terms of K-complexity. We show that there exists a limited number of graphs on *n* nodes that can be easily described to a machine using a constant number of bits. These graphs, which will be known as *basic graphs*, are then used for studying the randomness of graphs in general. After presenting an innovative methodology for distinguishing between random and non-random graphs, a novel algorithm is introduced to measure an upper bound for the K-complexity of graphs. We show that the algorithm is able to capture properties that make a graph non-random. Additionally, the applications of this algorithm in the context of evaluating graph compression methods is discussed.

## 2. Basic Graphs

In this section, we look for the least complex graphs possible with *n* vertices. Throughout this paper, it is assumed that the vertices of an *n*-node graph are labeled from 1 to *n*, and that the value of *n* is known to the machine. We first provide the definition for randomness deficiency, and then basic graphs are introduced as the easiest graphs to describe.

**Definition** **1** ([2] (Def. 6.4.2))**.** *A labeled graph G on n nodes has a randomness deficiency of at most δ(n), and is called δ(n)-random, if it satisfies*

C(E(G)|n,δ)≥n(n−1)/2−δ(n),

*where C(E(G)|n,δ) shows the K-complexity of the binary sequence of G’s adjacency matrix given n and δ.*

**Definition** **2** (basic graphs)**.** *A graph is basic if given n, its Kolmogorov complexity is of O(1). In other words, basic graphs have a random deficiency of n2−O(1).*

An example of a basic graph is the complete graph. If *n* is known, the entire graph can be described by simply stating that it is a complete graph, using a constant number of bits. The number of possible basic graphs is limited, as stated by the following theorem.

**Theorem** **1.** *The number of basic graphs is of O(1).*

**Proof of Theorem** **1.** It is known that a fraction of at most $1/2^{\delta (n)}$ of all labeled graphs on *n* nodes is δ(n)-random [2] (Lemma 6.4.1). Consequently, a fraction of at most 1/2n2−O(1) of the 2n2 possible labeled graphs on *n* nodes is basic. Therefore, the number of possible basic graphs is at most $2^{O(1)}$, which is a constant number.    □

To provide the conditions under which a graph is basic, we use the following definition.

**Definition** **3** (shift coefficient of a graph)**.** *The shift coefficient of a graph is the minimum positive number of circular shifts that needs to be applied to its node labels in order to reach a graph with the same adjacency matrix again. We use S(G) to show the shift coefficient of graph G.*

Notice that the direction in which the circular shift is applied to the labels is not important. This is because if we reach the same graph with a number of shifts in one direction, applying the same number of shifts in the opposite direction will also provide us with the same graph. Additionally, the maximum shift coefficient possible for a graph is *n*, as *n* shifts will provide us with the same graph again. The following lemma provides us with better insight into the possible values of the shift coefficient for a graph.

**Lemma** **1.** *The shift coefficient of a graph G with n nodes is always a divisor of n.*

**Proof.**  It can be seen that *n* circular shifts will always provide us with the same graph. If S(G)=n, the claim of the lemma holds. If not, then we must have S(G)<n. Consider *q* and *r* to be the quotient and remainder of dividing *n* by S(G), respectively. Notice that, as both qS(G) and *n* circular shifts will result in the same graph, then applying r=n−qS(G) circular shifts should also result in the same graph. However, we have 0≤r<S(G). *r* cannot be a positive value because if it was, it would contradict with the definition of S(G). Therefore, we must have r=0, and S(G) should be a divisor of *n*.    □

**Figure 1:**
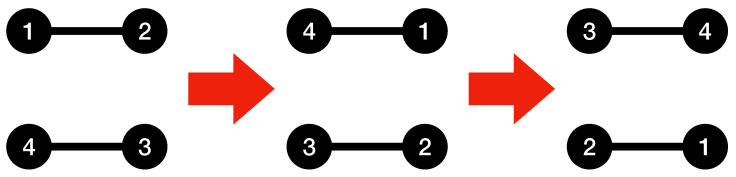
A graph with a shift coefficient of 2. It can be seen that we have the same graph after two circular shifts in the node labels.

Figure 1 provides an example of finding the shift coefficient for a graph.

We should point out the similarities between the definition of the shift coefficient and that of circulant graphs [21]. Circulant graphs can be defined as graphs with a shift coefficient of 1.

We are now equipped to state and prove the following theorem.

**Theorem** **2.** *A graph G with n nodes is basic if and only if it satisfies the following two conditions when given n.*

*1.*  *It has a shift coefficient with O(1) Kolmogorov complexity.*
*2.*  *The first S(G) columns of its adjacency matrix have a Kolmogorov complexity of O(1).*

**Proof of Theorem** **2.** Assume that graph *G* satisfies the two conditions. This graph can be described only by representing the connections of its first S(G) vertices. The connections of other vertices can easily be inferred by shifting the first S(G) nodes, based on the definition of shift coefficient. Additionally, describing the connections of the first S(G) nodes is of O(1) complexity. Therefore, representing S(G) and the connections is also of O(1) complexity; hence, the constant Kolmogorov complexity of the whole graph.

We prove the second side of the theorem by contradiction. Suppose there exists a graph *G* with O(1) Kolmogorov complexity that does not satisfy one of the two conditions of the theorem. Therefore, either the shift coefficient or the first S(G) columns of the adjacency matrix of the graph have a Kolmogorov complexity larger than O(1). However, the graph’s complexity is of O(1). This is not possible because both S(G) and the first S(G) columns can be inferred from the graph, and must have O(1) complexity themselves. As a result, a graph cannot be basic unless it satisfies both conditions of the theorem.    □

**Corollary** **1.** *The complement graph of any basic graph is also a basic graph.*

**Proof of Corollary** **1.** The description of any basic graph can be followed by an instruction of length O(1) to complement the graph. Therefore, the complement of a basic graph can be considered a basic graph itself.    □

**Corollary** **2.** *Isomorphisms of basic graphs are of at most $O(n\log_{2}n)$ K-complexity.*

**Proof of Corollary** **2.** If a graph is an isomorphism of a basic graph, it can be represented in the following manner. Firstly, one can describe the basic graph using O(1) bits. Additionally, the relabeling of each node can be described using at most $\left\lceil \log_{2}n\right\rceil$ bits that describe the new label of that node. Therefore, the relabeling of all nodes can be described using at most $n\lceil \log_{2}n\rceil$ bits. Consequently, the whole graph can be described using at most $n\left\lceil \log_{2}n\right\rceil +O\left(1\right)$ bits.    □

Theorem 2 provides us with a powerful tool to identify basic graphs. Many graphs are easily ruled out from being a basic graph using the first condition, as the shift coefficient can easily be calculated for any graph. The second condition is harder to check, as not all binary sequences with constant Kolmogorov complexity are known. However, we are still able to provide a list of graphs that fulfill both conditions and are therefore basic graphs. Table 1 shows some of the graphs that fulfill the conditions of Theorem 2. It must be noted that Table 1 only provides some examples of basic graphs. As their exact number is not known, there may exist other basic graphs. Therefore, we should consider the set of basic graphs used throughout this paper as a subset of all possible basic graphs (The problem of identifying all basic graphs remains open).

Notice that even though the definition of shift coefficient can usually help us in identifying basic graphs, it may not add any additional information for some cases. Based on Lemma 1, for a graph with *n* nodes, the possible set of shift coefficients consists of *n* and its divisors. Therefore, if *n* is prime or the set of its divisors has O(1) members, then all possible shift coefficients of the graph are of O(1) K-complexity. For these graphs, the shift coefficient condition does not provide us with any additional information about the K-complexity of the graph and we must only look at the connections between nodes.

## 3. Random and Non-Random Graphs

In this section, we distinguish between random and non-random graphs, and provide a methodology to detect which graphs are random and which are not.

**Definition** **4** (random graphs)**.** *A graph is a random or incompressible graph if it cannot be described to a universal Turing machine using less than n2 bits. In other words, a random graph is a graph with a randomness deficiency equal to zero. Any other graph will be called non-random or compressible.*

It is interesting to search for the maximum possible number of non-random graphs that exist on *n* nodes. Notice that in order for a graph to be considered non-random, it should be able to be described using n2−1 bits or less. Using n2−1 bits, one can index at most 2n2−1 graphs. Therefore, a fraction of at least half of all possible graphs will remain to be indexed using n2 bits or more. Consequently, it can be said that at least half of all possible graphs with *n* nodes are random graphs.

The following definition will aid us in our search for the properties of non-random graphs.

**Definition** **5** (basic subgraph)**.** *A subgraph g of graph G is called a basic subgraph if g is a basic graph given its number of vertices k, and an appropriate relabeling of its nodes using the labels {1,…,k}.*

We will now prove an important theorem about random graphs using the incompressibility method [22].

**Theorem** **3.** *Random graphs do not contain any basic subgraphs with more than $2\log_{2}n+4$ vertices.*

**Proof of Theorem** **3.** Suppose that there is a random graph that includes a basic subgraph of size $2\log_{2}n+5$ or larger. We provide a method to represent such a graph using fewer than n2 bits, which proves the theorem by contradiction.

Suppose that a graph with *n* nodes contains a basic subgraph with *k* nodes. To describe a basic subgraph of order *k*, we first need to specify its size, *k*. We can do this using $\left\lceil \log_{2}n\right\rceil$ bits. Additionally, we need to describe the nodes that form this subgraph. The nodes of the graph can be labeled using $\left\lceil \log_{2}n\right\rceil$ bits. Therefore, $k\lceil \log_{2}n\rceil$ bits are needed to specify the *k* nodes of the basic subgraph. Notice that for some basic graphs, the order in which the label of these nodes are listed may be of importance. For example, if the basic graph is a cycle, the labels of its nodes need to be listed in the order of their connection. Additionally, O(1) bits are needed to specify the basic graph at hand. Using this method, instead of using k2 to represent the edges of a basic subgraph, we are using $k\left\lceil \log_{2}n\right\rceil +\left\lceil \log_{2}n\right\rceil +O\left(1\right)$ bits. As the constant term can be ignored as *n* grows large, we can use the following inequality to determine the values of *k* for which this method of representation provides us with a code with less than n2 bits.

(1) k2>(k+1)⌈log2n⌉

On the other hand, the following inequality holds for the right hand side of Equation (1).

(2) $$\left(k+1\right)\left\lceil \log_{2}n\right\rceil \leq \left(k+1\right)\left(\log_{2}n+1\right)$$

Therefore, we search for values of *k* that satisfy

(3) k2>(k+1)(log2n+1).

Solving the quadratic inequality of Equation (3) provides us with the following positive integer values for *k*.

(4) $$k>2\log_{2}n+4$$

Therefore, a random graph cannot have a basic subgraph larger than $2\log_{2}n+4$. Otherwise, it can be described with the explained method and have a positive randomness deficiency.    □

Notice that the graph representation method used in the proof of Theorem 3 results in descriptions with the same lengths for isomorphic graphs. However, there are techniques that can be used to reduce the code word length even more for some graphs, depending on their node labeling. For example, we can use techniques such as variable-length encoding and gap encoding [18] to describe the node labels of a basic subgraph more efficiently. An optimal method for describing the node labels of a basic subgraph should also use a code of length O(1) when all the nodes of a graph form a basic graph, and the node ordering is not important (such as a complete graph). Throughout the rest of this paper, we will assume that we use one of such techniques to code a set of ordered node labels. The function label_codin(L) returns a coded version of its input *L*, which is an ordered node list. Notice that according to the method used in the proof of theorem 3, the code length for a list of *k* nodes should not be longer than $\left(k+1\right)\left\lceil \log_{2}n\right\rceil$ bits.

Because of their importance in the following discussions, we define the term “semi-basic graphs” as below.

**Definition** **6** (semi-basic graphs)**.** *A graph on n nodes is called semi-basic if it contains at least one basic subgraph with more than $2\log_{2}n+4$ nodes.*

Theorem 3 states an important feature of random graphs. Additionally, it also specifies an important family of non-random graphs. This theorem acts as the basis of our method for detecting non-randomness in graphs.

## 4. Kolmogorov Graph Covering

As discussed in the previous section, semi-basic graphs provide a reliable measure of detecting non-randomness in graphs. Therefore, we use these graphs as the basis of our proposed method for calculating an upper bound on the Kolmogorov complexity of graphs. The proposed algorithm, Kolmogorov graph covering, tries to cover its input graph with basic graphs of size $2\log_{2}n+5$ or larger; thus, reducing the complexity of the input graph. Algorithm 1 demonstrates Kolmogorov graph covering.
**Algorithm** **1.** The Kolmogorov graph covering algorithm.
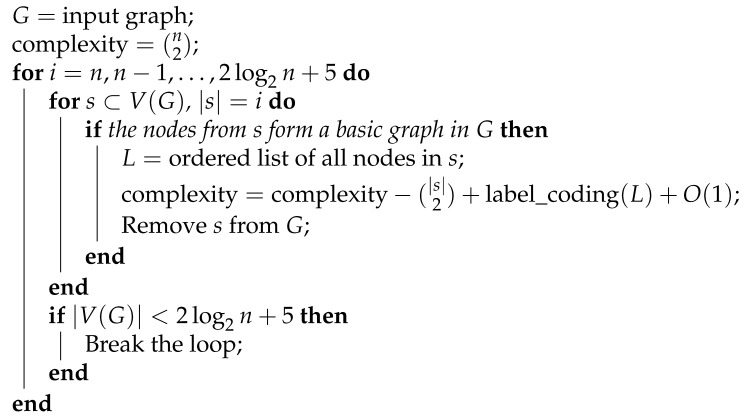


In Algorithm 1, V(G) represents the set of all vertices present in graph *G*.

The essence of Algorithm 1 is that it finds the biggest basic subgraphs of its input graph, and uses those subgraphs to reduce the complexity of its input if possible. If it encounters a basic subgraph larger than $2\log_{2}n+4$, the algorithm uses the explained method for describing basic subgraphs to reduce the complexity of the input graph. Afterwards, it will remove the nodes of the basic subgraph from the original graph, and will search for other basic subgraphs. The reason for this is to avoid having any duplicate edges when covering the graph with basic subgraphs. Ultimately, the algorithm outputs its estimation for the upper bound of the K-complexity of its input graph.

The graph coding method corresponding to the complexity calculated in Algorithm 1 is as follows. First, the basic subgraphs within the original graph are presented, using O(1) bits each. Then, the nodes that are part of these subgraphs are specified in order. Finally, the code ends with the simple adjacency matrix representation of unspecified edges in a lexicographical order. Note that Algorithm 1 has a very high computational complexity. This is because it basically goes through all possible subgraphs larger than $2\log_{2}n+4$. Consequently, Algorithm 1 is not suitable to be used as a graph compression algorithm itself. However, if computation time was to be ignored, Algorithm 1 would have been a powerful graph compression algorithm.

The main purpose of Algorithm 1 is to act as a tool for calculating an upper bound on the K-complexity of graphs. This can have many applications, especially in graph compression. One of the most important application scenarios of Algorithm 1 is to provide an evaluation metric for graph compression algorithms. We can test the performance of a graph compression algorithm for a particular graph by inputting that graph into both the compression algorithm and Algorithm 1, and then compare the output length of the compression algorithm with the complexity estimation that Algorithm 1 has provided. If the compression algorithm has a larger output length than the output of Algorithm 1, it can be inferred that the input graph could have been compressed more efficiently. Otherwise, it can be said the compression algorithm is performing well in compressing that particular graph, and it has found a better upper bound on the K-complexity of the input graph. Notice that this method is only valid for assessing a compression method when the method performs the compression solely based on its input graph, and does not assume a probability distribution on an ensemble of graphs.

It is not claimed that Algorithm 1 provides the most compressed version of the graph possible. One of the most important examples of this is sparse graphs. For sparse graphs, the adjacency list representation of graphs may provide us with a lower K-complexity for these graphs. Similarly, very dense graphs may also be represented using fewer bits than what Algorithm 1 provides us with by only listing the missing edges. The main claim is that semi-basic graphs can be used to distinguish between random and non-random graphs, and that Algorithm 1 provides us with a relatively good upper bound for the K-complexity of different graphs.

It can be seen that Algorithm 1 is ignoring the simplicities that may exist in the connection between pairs of basic subgraphs after they are found. The algorithm is assuming that the adjacency matrix representation is used to show the connections between the basic subgraphs. However, these connections may be further simplified and be used to reduce the K-complexity of the whole graph even more. To this end, the following algorithm is presented as an optional expansion to Algorithm 1.

**Figure 2:**
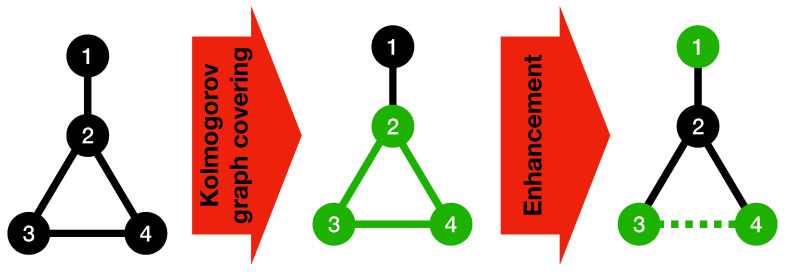
Enhanced Kolmogorov graph covering. The first step is a basic Kolmogorov graph covering where nodes 2,3, and 4 have been detected as a basic graph (complete graph). To describe the connections between node 1 and the complete graph, the enhancement algorithm has detected another basic graph (null graph) with nodes 1, 3, and 4 by considering the edge between nodes 3 and 4 to be non-existent. Notice that the aim of this figure is to illustrates the method used in Algorithm 2, and does not necessarily reduce the complexity of this particular graph (because of its small size).

Algorithm 2 can be run on the input graph after Algorithm 1 to reduce the complexity of the connections between the basic subgraphs found by the Kolmogorov graph covering algorithm. Because the edges within each basic subgraph have once been taken into account by Algorithm 1, there is no need to list them again. Instead, those edges can be chosen in a way that they contribute towards building a bigger basic subgraph using the nodes of pairs of basic subgraphs found by Algorithm 1, which will result in a lower complexity for the graph. The condition checked in the if-statement of Algorithm 2 is to ensure that using the basic graph representation for the selected vertices will result in a reduction in the K-complexity of the graph. Figure 2 illustrates the procedure of Algorithm 2 using a simple example.
**Algorithm** **2.** Kolmogorov graph covering enhancement add-on.
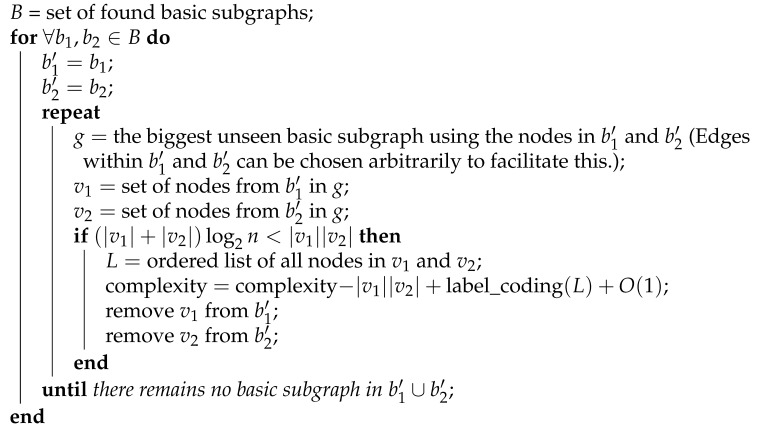


We discussed the evaluation of the performance of graph compression algorithm on single graphs, but we were also interested in assessing the compression algorithm in general. To achieve this, the following method is proposed. Instead of using a single graph, one can have a set of *k* graphs $\left\{G_{1},G_{2},\ldots ,G_{k}\right\}$. Ideally, we need to have all possible graphs on *n* nodes in this set to completely capture the performance of the compression algorithm, but this may not be feasible. Let $L_{A}\left(G_{i}\right)$ and $K^{\prime }\left(G_{i}\right)$ return the length of the compressed version of $G_{i}$ using graph compression algorithm *A*, and the estimated Kolmogorov complexity of $G_{i}$ using Kolmogorov graph covering, respectively. We can use the following formula as a metric to evaluate the performance of the compression algorithm.

(5) $$M\left(A\right)=\frac{1}{k}\sum_{i=1}^{k}K^{\prime }\left(G_{i}\right)-L_{A}\left(G_{i}\right)$$

M(A) in Equation (5) acts as a variable that can show the performance of the graph compression algorithm *A*. If M(A) is a positive value, it means that the compression algorithm provides a shorter code length than the complexity values returned by Algorithm 1 on average. If M(A) is a negative value, it means that the average code length for graphs present in the set can be less than what *A* achieves. In all, M(A) can be used as a metric to assess the performance of different graph compression methods and compare them together.

## 5. Simulation Results and Discussion

The Kolmogorov graph covering was simulated using MATLAB. In this section, some applications of this algorithm are explored and simulation results are presented.

We built a pool of basic graphs to be used in our code for the Kolmogorov graph covering algorithm. As mentioned earlier, we cannot be sure about the number of possible basic graphs. Therefore, we are only using a subset of them in our code. The following graphs and their complements are included in our pool of basic graphs: complete graph, cycle graph, ladder rung graph, and one-edge graph. These graphs are all present in Table 1. It is interesting to observe that even with such a limited number of basic graphs, one can detect elements of non-randomness in the input graphs.

The reported results for the Kolmogorov graph covering algorithm contain an extra term, of O(1). This term is added to remind the reader of the O(1) bits that need to be used for representing the discovered basic subgraphs. Even though the exact number of bits that are going to be used for representing basic graphs cannot be precisely known, and may vary based on the coding technique, it does not grow with *n*.

### 5.1. Traditional Graph Representation Methods

In this section, the results of our method for estimating the complexity of graphs is compared with code lengths from traditional graph coding techniques, namely adjacency matrix and adjacency list.

**Figure 3:**
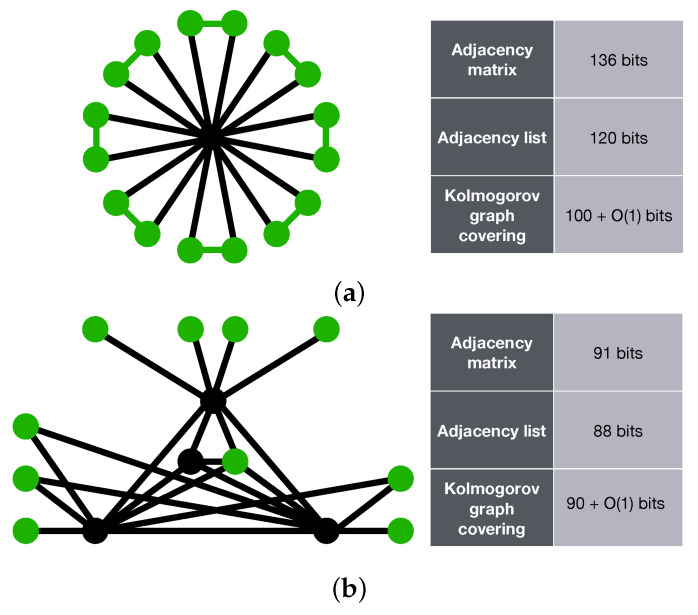
Two sample graphs shown with their K-complexity estimates using three methods: adjacency matrix, adjacency list, and Kolmogorov graph covering. (**a**) Example graph 1; (**b**) Example graph 2. The green nodes and edges show the basic subgraphs found by the Kolmogorov graph covering algorithm. The basic subgraphs in (**a**,**b**) are a ladder rung graph and a null graph, respectively.

Figure 3 illustrates two sample graphs, alongside with their K-complexity estimate using three approaches. Adjacency matrix and adjacency list have been used as coding methods that will provide us with an upper bound on the K-complexity of graphs. The Kolmogorov graph covering algorithm has also been run on both graphs. It can be observed that Kolmogorov graph covering has presented a much lower K-complexity for the first graph compared to its adjacency list and adjacency matrix representation. For the second graph, all three approximations are returning nearly the same result.

### 5.2. Zachary’s Karate Club

**Figure 4:**
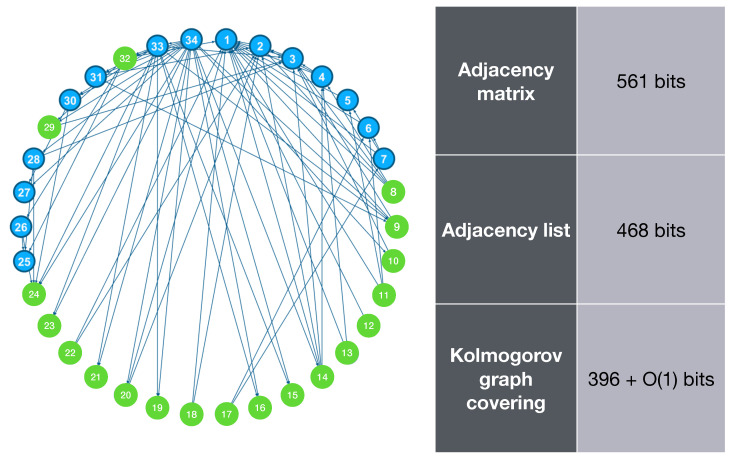
Zachary’s karate club graph, along with its K-complexity estimation using adjacency matrix, adjacency list, and Kolmogorov graph covering. The nodes in green are detected by the algorithm as forming a basic subgraph (null graph). Original graph picture source: https://www.learndatasci.com/tutorials/k-means-clustering-algorithms-python-intro/ accessed on 11 November 2021.

Zachary’s karate club is a graph based on the social interactions of the members of a karate club that was studied by Wayne W. Zachary [23]. This graph is often used as a dataset in studies related to network science. In this section, we apply the Kolmogorov graph covering algorithm to this 34-node graph and measure its complexity. The result is illustrated in Figure 4.

Figure 4 shows that the complexity of this graph is much less than its adjacency matrix and adjacency list representations, and this is done by detecting only one basic subgraph.

### 5.3. Existing Graph Compression Algorithms

**Figure 5:**
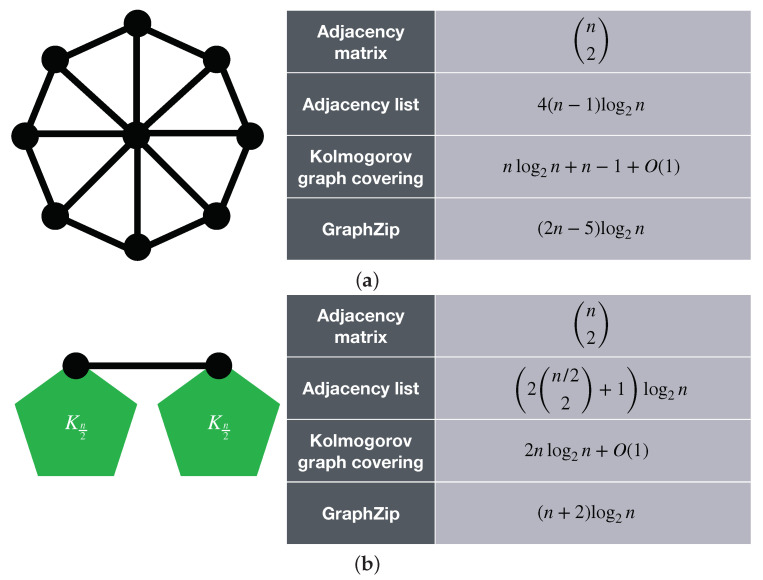
Comparing the results of Kolmogorov graph covering and GraphZip. (**a**) shows a wheel graph of size *n*, in which Kolmogorov graph covering detects a ring graph of size n−1, and GraphZip detects a clique of size 3 and (n−3)/2 cliques of size 2. (**b**) illustrates two complete graphs of size n/2 connected by a single edge. In this case, the enhanced Kolmogorov graph covering detects two complete graph of size n/2, and a null graph of size n−2 to describe the connections between the two complete graphs. GraphZip detects two cliques of size n/2.

As stated, one important application of the Kolmogorov graph covering algorithm is to test and evaluate existing graph compression methods. In this section, we evaluate the performance of GraphZip [24] on a number of graphs. GraphZip was introduced in 2018 as a lossless graph compression method for sparse graphs. This graph compression method identifies the biggest cliques within the input graph, and defines a new pseudo vertex for each of them. Ultimately, it represents the graph using an adjacency list approach, by considering the membership of a node in a clique as an edge between that node and the clique’s pseudo vertex. Figure 5 shows the results of applying Kolmogorov graph covering and GraphZip to two example graphs. It can be seen that one graph Kolmogorov graph covering provides a lower estimate for the K-complexity of the graph, whereas for the other one, GraphZip provides a shorter code length. This means that the graph of Figure 5a could have been compressed more efficiently, but GraphZip is already performing well for the graph of Figure 5b. It can also be observed that for both cases, the complexities are from the same order of magnitude. These examples show how the Kolmogorov graph covering can be used to evaluate the performance of lossless graph compression algorithms on single graphs.

### 5.4. Future Work

The authors believe that the findings of this paper can be further explored in the future. One of the suggested areas that can be further studied is obtaining better insight into the structure of basic graphs and their numbers. Additionally, using more efficient techniques for representing node labels of a basic subgraph will result in a better upper bound for the K-complexity of graphs. Moreover, finding a faster implementation for the Kolmogorov graph covering algorithm will have potential applications in graph compression.

## 6. Conclusions

In this paper, the complexity of graphs was explored by using Kolmogorov complexity. Basic graphs were introduced as the least complex graphs possible. It was shown that because of their properties, basic graphs can be used as a tool to distinguish between random and non-random graphs. Based on this observation, the Kolmogorov graph covering algorithm was presented as a method to estimate an upper bound for the K-complexity of its input graph. We also discussed how the algorithm may be unable to capture some non-randomness in the input graph. To overcome this, an extension to the algorithm was proposed. Additionally, we showed how the Kolmogorov graph covering can be used to evaluate the performance of graph compression algorithms. Finally, the results of applying the algorithm on a number of example graphs was presented, and we discussed how these results can be interpreted.

## Figures and Tables

**Table 1:** Examples of basic graphs. Notice that the number of nodes used in the example graphs is for visualization purposes only, and it can easily be extended to any number.

Name	Shift Coefficient	Connection Description	Example Graph
Complete graph	1	Each node connects to all other nodes.	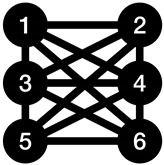
Cycle graph	1	Each node connects to the nodes directly before and after itself.	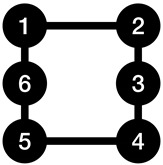
Star polygon graph with a density of 2	1	Each node connects to the nodes directly two positions before and after itself.	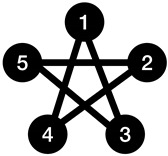
Ladder rung graph	2	Odd nodes connect to the node after, even nodes connect to the node before.	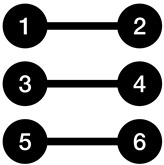
One-edge graph	*n*	There is an edge between the first two nodes.	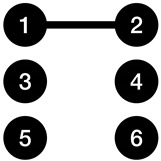

## Data Availability

For the experiments in this paper, we used a publicly available dataset called Zachary’s karate club, which can be accessed at http://konect.cc/networks/ucidata-zachary/ accessed on 11 November 2021.
